# Effect of Surgical Delay on Intracapsular and Intertrochanteric Femoral Fractures on Mortality and Adverse Events: A Systematic Review and Meta-Analysis

**DOI:** 10.7759/cureus.109829

**Published:** 2026-05-28

**Authors:** Thea Difesa

**Affiliations:** 1 General Surgery and Orthopaedics, Mater Dei Hospital, Malta, MLT

**Keywords:** femoral fracture, intertrochanteric, intracapsular, morbidity, mortality, surgical fixation, surgical timing

## Abstract

Overall, the worldwide incidence of hip fractures is increasing due to an aging population. These fractures contribute significantly to morbidity and mortality, placing a substantial burden not only on the healthcare system but also on society. This study aims to clarify the impact of surgical timing on clinical outcomes in femoral neck fractures by addressing a crucial modifiable factor in patient care. The primary focus was to evaluate the impact of time to surgery from sustained injury on mortality. The secondary objectives were to evaluate the effect of time to surgery on other adverse outcomes, including postoperative complications and hospital length of stay.

Using the PICOS (population, intervention, comparison, outcome, and study design) framework, the study focused on 30-day and inpatient mortality as the primary outcomes. A literature search, a sub-meta-analysis, and a narrative synthesis were used to delve deeper into this somewhat elaborate and inconclusive topic. Most patients experienced surgical delay, and the sub-meta-analysis showed a nonsignificant trend toward reduced inpatient mortality with early surgery, whereas the narrative synthesis revealed mixed findings. Complication rates and hospital stay outcomes also varied, underscoring the complex interplay between surgical timing, patient comorbidities, and clinical outcomes. Prioritizing adequate medical optimization preoperatively remains primitive and may contribute more to improved outcomes than surgical timing alone.

## Introduction and background

The incidence of hip fractures is increasing worldwide, mainly due to an aging population, with projections estimating a substantial rise in incidence over the coming decades [1]. Hip fractures are associated with high morbidity and mortality, threatening patients’ mobility, independence, and, thus, quality of life, with reported 30-day mortality rates of 5%-10% and one-year mortality exceeding 25% in some populations [2]. In addition to clinical consequences, around 30% of patients show some extent of permanent disability after sustaining a hip fracture [3,4]. The economic impact places a significant burden on the healthcare system, which is further exacerbated by complications arising from hip fractures. Given the significant morbidity and deterioration of functional capacity associated with these fractures, it is crucial to identify any modifiable factors that may improve clinical outcomes and enhance quality of life [5].

Timely surgical intervention has long been considered a key factor influencing prognosis in hip fracture patients; in fact, several studies suggest performing surgery as soon as possible, yet the optimal timing for surgery remains debated [6]. Menninger et al. [7] concluded that performing surgery within six hours significantly reduces the risk of avascular necrosis, leading to superior outcomes compared to surgeries conducted beyond this critical time frame. Saul et al. [8] concurred with this finding, reporting that delayed treatment led to prolonged hospital stays; however, they also found that postponing surgery was associated with a reduction in non-surgical complications, such as electrolyte imbalances, chest infections, and anemia. In contrast, a 32-year retrospective clinical study [9] found no significant clinical correlation between the timing of intracapsular fracture fixation and adverse events. Similarly, the HIP ATTACK trial [10], an international randomized controlled trial, did not find that accelerated surgery led to improved clinical outcomes or a reduced complication rate.

The relationship between time to surgery and clinical outcomes remains uncertain. While some studies suggest that earlier intervention may reduce complications and mortality, others report no significant association or even propose that short delays may allow for medical optimization without adversely affecting outcomes. These inconsistencies are compounded by substantial heterogeneity in study design, patient populations, and definitions of surgical delay, with timing frameworks ranging from ultra-early intervention to broader thresholds such as surgery within 24-48 hours [7-10]. As these approaches reflect distinct clinical questions and are not directly comparable, this review focuses specifically on the commonly used threshold of 48 hours from hospital admission, consistent with prevailing clinical practice and the definitions applied in the included studies.

Current clinical guidance from the National Institute for Health and Care Excellence (NICE) and the American Academy of Orthopaedic Surgeons (AAOS) emphasizes early surgical intervention following hip fracture, defined from the time of hospital admission, with NICE recommending surgery on the day of or the day after admission and AAOS recommending surgery within 24-48 hours [11,12]. However, these recommendations are based on evidence that is not entirely consistent, and the optimal timing threshold remains debated. By synthesizing the available evidence, this review seeks to clarify whether earlier operative intervention within 48 hours of admission is consistently associated with improved inpatient mortality and clinical outcomes, while accounting for the methodological limitations and variability inherent in the existing literature.

Aim

This study examines the association between time to surgical intervention and clinical outcomes in patients with intracapsular or intertrochanteric hip fractures, with time to surgery consistently defined as the interval between hospital admission and operative management.

The primary outcome was inpatient mortality. Secondary outcomes included 30-day and one-year mortality, as well as postoperative complications and length of hospital stay, providing a broader context regarding patient outcomes and healthcare utilization.

The principal comparison evaluated patients undergoing surgery within 48 hours of admission versus those experiencing delays beyond this threshold, thereby operationalizing surgical timing as a categorical exposure aligned with current evidence-reporting practices.

This study critically evaluates the strength and consistency of these observed associations without assuming causality, recognizing the potential impact of confounding factors and variability in perioperative care pathways. By maintaining a consistent temporal definition and a clearly defined outcome hierarchy, the analysis aims to provide a methodologically robust interpretation of the relationship between surgical timing and patient outcomes.

## Review

Methods

The research question was formulated using the PICOS (population, intervention, comparison, outcome, and study design) framework to define the inclusion criteria [13]. The target population included adults aged 60 years or older who underwent orthopedic surgical intervention after sustaining either an intracapsular or intertrochanteric hip fracture (Table 1). Patients were grouped according to surgical timing as reported in the included studies, which consistently defined timing relative to hospital admission. Early surgery was operationalized as surgery performed within 48 hours of admission, while delayed surgery was defined as surgery beyond this threshold. This definition reflects the timing metrics used across the included studies and is broadly consistent with guideline recommendations that emphasize early intervention following admission, although NICE and AAOS do not prescribe an identical fixed threshold [11,12]. Therefore, surgical delay referred to patients who underwent surgical intervention more than 48 hours after admission. Only English-language papers published after January 1, 2000, including randomized controlled trials and prospective or retrospective observational studies, were included. Pediatric patients, case reports, and other systematic reviews were excluded from the study.

**Table 1:** PICOS criteria PICOS: Population, intervention, comparison, outcome, and study design. Source: Ref. [13].

Population	Adults aged 60 years or older who sustained an intracapsular or intertrochanteric hip fracture
Intervention	Any orthopedic surgical intervention
Comparison	Undergone surgery within 48 hours of admission
Outcomes	Mortality, length of stay, postoperative complications
Study design	Randomized controlled trials, prospective and retrospective observational studies

The systematic review protocol was registered with the International Prospective Register of Systematic Reviews (PROSPERO) to ensure transparency and reduce the risk of bias (registration number: CRD420251061161). A literature search was conducted using the following Boolean search strategy: (“femoral neck fracture” OR “hip fracture”) AND (“timing” OR “delay” OR “early” OR “late” OR “time to surgery”) AND (“fixation” OR “surgical fixation” OR “surgery”) AND (“outcome” OR “morbidity” OR “mortality” OR “functional recovery” OR “complications”). The literature search was conducted through Ovid MEDLINE, the Cochrane Library, Embase, and PubMed, and filters reflecting the inclusion criteria described above were applied. The search was conducted during the first week of January 2025. The filters applied included studies published between 2020 and 2024, English-language publications, human studies, and study designs including randomized controlled trials, prospective studies, retrospective studies, and observational studies. Additionally, the reference lists of all systematic reviews encountered during the search were screened for possible studies that matched the inclusion criteria of this study. Articles identified in each database were recorded in an Excel sheet (Microsoft Corp., Redmond, WA), including the dates on which the searches were conducted.

The total number of records identified from each database was recorded, and the cumulative total across all databases was calculated. The Preferred Reporting Items for Systematic Reviews and Meta-Analyses (PRISMA) flow diagram outlines the study selection process [14]. After removing duplicates (n = 316), 609 unique records remained for screening. These were assessed based on titles and abstracts, leading to the exclusion of studies that did not meet the inclusion criteria. Full texts of the remaining 273 articles were reviewed in detail. Of the 273 reports assessed for eligibility, 264 were excluded during full-text review, primarily due to ineligible fracture type (n = 67), inappropriate comparators (n = 92), absence of surgical timing data (n = 89), and other reasons (n = 16). The “other reasons” category (n = 16) comprised studies in which surgical timing was not the primary exposure of interest, including comparisons based on surgical approach, service organization (e.g., out-of-hours care), or inter-hospital variation; studies with substantial confounding or non-comparable designs; investigations limited to niche or non-representative populations (e.g., COVID-19 cohorts or age-restricted subgroups); and studies with outcomes or interventions not aligned with the present research question. Ultimately, nine studies met all eligibility criteria and were included in the systematic review. All records were screened manually; no automation tools were used. As the included studies were predominantly observational, methodological quality was assessed using the Newcastle-Ottawa Scale (NOS) [15], which evaluates study selection, comparability, and outcome domains. Each study was independently scored by two reviewers. Discrepancies between reviewers were resolved through discussion to reach consensus, and the agreed-upon scores were used in the final analysis. Overall NOS scores ranged from 7 to 9, indicating generally moderate to high methodological quality. However, these ratings should be interpreted with caution, as observational studies examining surgical timing remain susceptible to residual confounding and bias not fully captured by the NOS framework.

A structured data extraction form was created using an Excel spreadsheet to systematically collect and organize information from the included studies. Key outcomes of interest were carefully identified and compiled, including study design, patient demographics, mortality, adverse events, length of stay, surgical timing, relevant statistical values and measures, and other pertinent data reported in each publication. The extracted information was subsequently examined using both a sub-meta-analysis to quantitatively assess specific outcomes and a narrative synthesis to qualitatively interpret trends and findings across studies. This dual approach ensured a thorough and well-rounded interpretation of the available evidence.

A sub-meta-analysis using Review Manager (RevMan, The Cochrane Collaboration, London, United Kingdom) [16] was conducted to determine whether the primary outcome demonstrated statistical significance. A random-effects model was used to account for expected variation between studies in populations, timing definitions, and methodology. Studies reporting inpatient mortality were included in the meta-analysis, and their data were pooled in RevMan. Other studies were excluded from the sub-meta-analysis, as the inpatient mortality was not included in their results. Heterogeneity, relative risk (RR), and statistical significance were assessed, and a forest plot was generated. For interpretation of the results, a significance level of p < 0.05 was set as the threshold for statistical significance. Furthermore, other mortality rates and a range of subsequent clinical outcomes were systematically explored through qualitative narrative synthesis. This allowed a more in-depth insight into each study individually, exploring the unique factors addressed in each and further identifying the impact of various elements on patient survival.

Results

**Figure 1:**
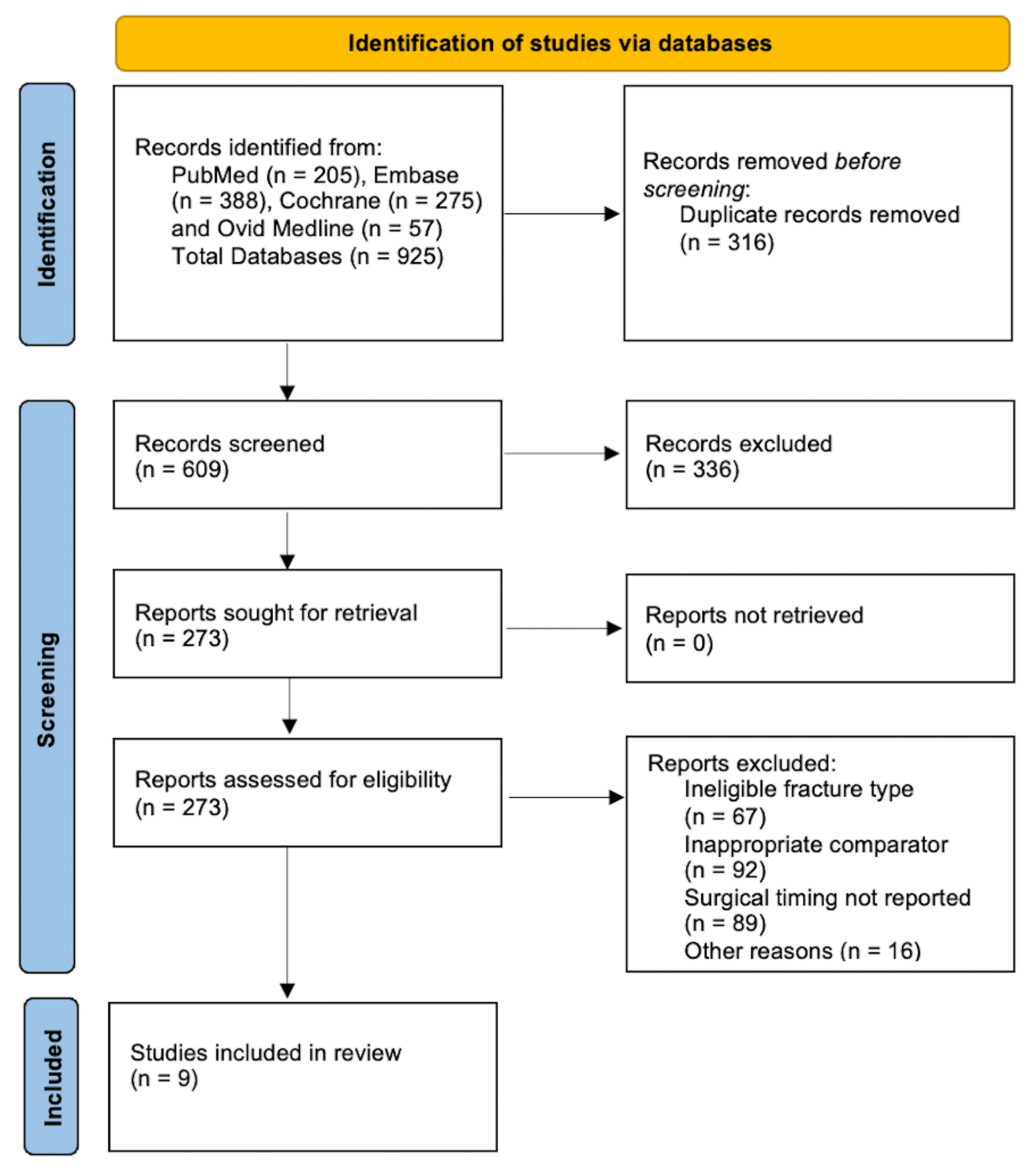
PRISMA flow diagram PRISMA flow diagram showing the breakdown of the screening and selection process of the included studies [14].

The primary literature search identified 925 records. After deduplication, 609 records were screened based on title and abstract. Of the 273 articles assessed for eligibility, nine observational studies were included in the final analysis [14]. The literature search and study selection process are shown in the PRISMA flow diagram (Figure 1).

All included studies had an NOS rating of 7 or higher, indicating a low risk of bias across the included studies (Table 2) [15].

**Table 2:** NOS table of results NOS rating breakdown by component for each paper, as assessed by both the primary and secondary reviewers [15]. NOS: Newcastle-Ottawa scale.

Reference	Selection (/4)	Comparability (/2)	Outcomes (/3)	Total NOS	NOS of the second reviewer
Librero et al. [6]	4	1	2	7	7
Kanthasamy et al. [9]	4	1	3	8	8
Lizaur-Utrilla et al. [17]	4	2	2	8	9
Paul and Issac [18]	4	1	3	8	8
Oakley et al. [19]	4	2	3	9	9
Daginnus et al. [20]	4	1	2	7	9
Shoda et al. [21]	4	2	1	7	9
Anthony et al. [22]	4	1	3	8	8
Patczai et al. [23]	4	1	3	8	9

Of the included studies, one was prospective, while the remaining eight were retrospective. The data collection period ranged from 1989 to 2020. A total of 129,016 patients with either femoral neck or intertrochanteric fractures who underwent surgical intervention were included. Of these, 16,548 patients (12.83%) underwent surgery within 48 hours and were classified as having no surgical delay, while 69,218 patients (53.65%) experienced delayed surgical intervention. For the remaining 33.52% of patients, insufficient data were available to determine surgical timing, limiting subgroup classification. These data are summarized in Table 3.

**Table 3:** Study descriptions This table presents the overview of all studies included in the systematic review, detailing the total number of patients and whether their surgeries were delayed or non-delayed [6,9,17-23].

Reference	Type of study	Data collection period	Fracture type	Total number of patients	Patients who were not experiencing surgical delay	Patients who were experiencing surgical delay
Librero et al. [6]	Retrospective observational	2002-2005	Intracapsular and intertrochanteric (IT)	52803	13147 (24.9%)	39656 (75.1%)
Kanthasamy et al. [9]	Retrospective observational	1989-2020	Intracapsular	2366	/	/
Lizaur-Utrilla et al. [17]	Prospective observational	2010-2014	IT and cervical	628	180 (28.66%)	448 (71.34%)
Paul and Issac [18]	Retrospective observational	2005-2009	Femoral neck and IT	144	58 (40.28%)	86 (59.72%)
Oakley et al. [19]	Observational cohort	2008-2010 and 2012-2014	Femoral neck	2541	863 (33.96%)	/
Daginnus et al. [20]	Retrospective observational	2010-2014	Intracapsular and IT	575	256 (44.52%)	/
Shoda et al. [21]	Retrospective observational	2007-2009	Intracapsular and IT	66893	/	28956 (43.29%)
Anthony et al. [22]	Retrospective cohort	2005-2010	Femoral neck and IT	176	104 (59.09%)	72 (40.91%)
Patczai et al. [23]	Retrospective observational cohort	2000-2008	Femoral neck	2890	1940 (67.13%)	/

Lizaur-Utrilla et al. [17] observed an inpatient mortality rate of 0.50% in patients without surgical delay and 1.10% in those with delayed surgery. Paul and Issac [18] reported inpatient mortality rates of 3.44% in the non-delayed group and 11.62% in the delayed group, while Librero et al. [6] found inpatient mortality rates of 6.83% in patients without surgical delay and 3.23% in those who experienced delayed surgery.

**Figure 2:**
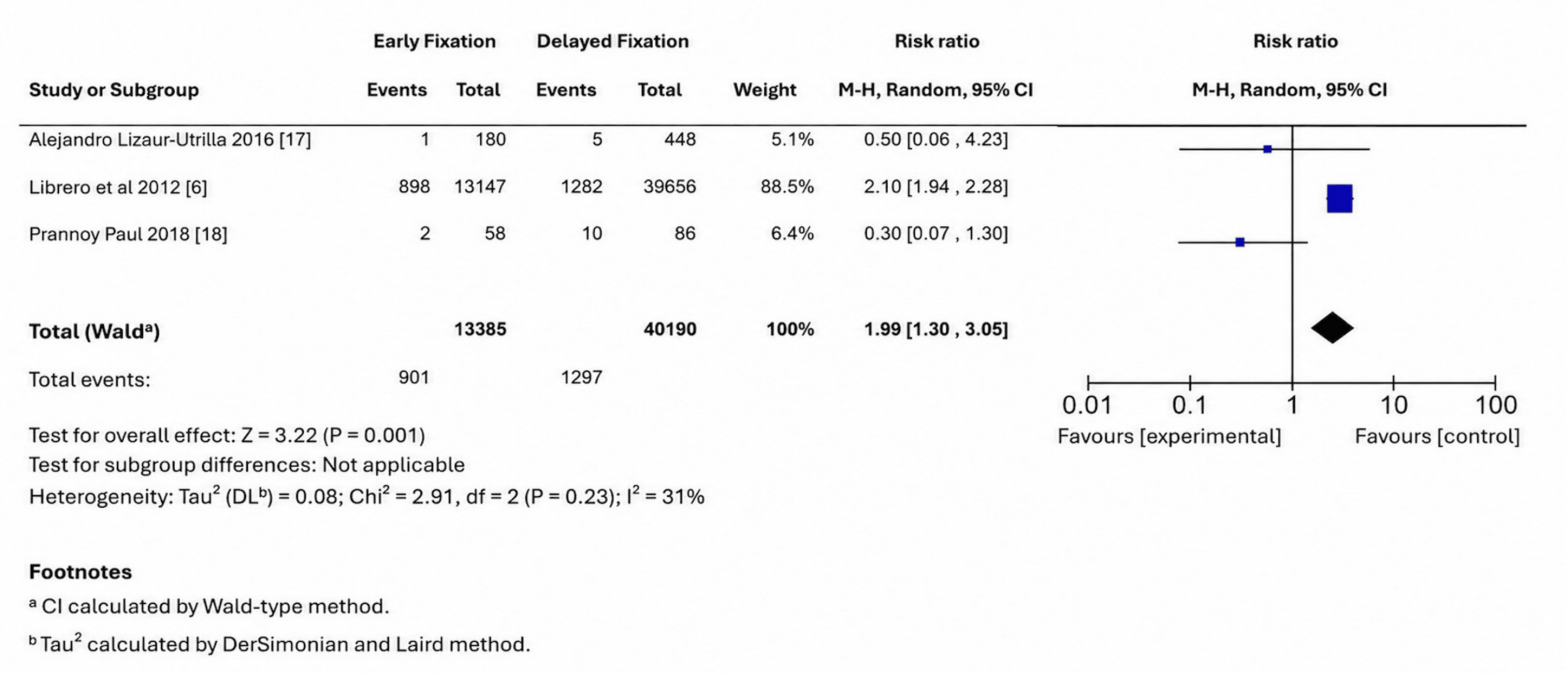
Forest plot Forest plot of the sub-meta-analysis [6,17-18]

A meta-analysis (Figure 2) was performed on studies reporting inpatient mortality rates, as highlighted in Table 4. A total of 53,575 patients were included, with 13,385 in the early surgery group and 40,190 in the delayed surgery group [6,17,18]. There were 901 events in the early surgery group and 1,297 in the delayed surgery group. The pooled risk ratio was 1.99 (95% CI: 1.30-3.05; p = 0.001), indicating a statistically significant association. Statistical heterogeneity was low to moderate (I² = 31%).

**Table 4:** Inpatient mortality rates Studies included in the sub-meta-analysis [6,17,18].

Reference	Inpatient mortality in those not experiencing surgical delay	Inpatient mortality in those experiencing surgical delay
Librero et al. [6]	6.83%	3.23%
Lizaur-Utrilla et al. [17]	0.5%	1.10%
Paul and Issac [18]	3.44%	11.62%

Oakley et al. [19] reported a 30-day mortality rate of 6% (52/863) in patients who underwent surgery within 36 hours, compared with 21% (66/314) in those operated on after 36 hours (p < 0.05). For one-year mortality, Oakley et al. [19] reported rates of 28.6% (196/863) in the early surgery group (<36 hours) and 42% (132/314) in the delayed surgery group (>36 hours) (p < 0.05). In contrast, Lizaur-Utrilla et al. [17] observed a three-month mortality rate of 5% in patients operated on within 48 hours and 7.3% in those whose surgery was delayed beyond two days; however, this difference was not statistically significant (p = 0.3777). For one-year mortality, Lizaur-Utrilla et al. [17] reported rates of 14.4% in the <48-hour group and 13.6% in the >48-hour group (p = 0.799). Both Oakley et al. and Lizaur-Utrilla et al. used the chi-square (χ²) test to determine statistical significance between groups (Table 5) [17,19].

**Table 5:** Comparison of mortality rates based on the timing of surgical intervention Source: Refs. [17,19].

Reference	Outcome	Timing of surgical intervention	Mortality (%)	P-value (χ²)
Oakley et al. [19]	30-day mortality	<36 hours	6% (52/863)	<0.05
		>36 hours	21% (66/314)	<0.05
	1-year mortality	<36 hours	28.6% (196/863)	<0.05
		>36 hours	42% (132/314)	<0.05
Lizaur-Utrilla et al. [17]	3-month mortality	<48 hours	5%	0.3777
		>48 hours	7.30%	0.3777
	1-year mortality	<48 hours	14.40%	0.799
		>48 hours	13.60%	0.799

With regard to secondary outcomes, Daginnus [20] reported a higher number of complications in patients undergoing early surgery (94 complications within 24 hours versus 14 complications after 24 hours). For length of stay, findings were inconsistent: Oakley et al. [19] found no statistically significant difference between early and delayed surgery (p = 0.236), whereas Lizaur-Utrilla et al. [17] reported a shorter overall hospital stay in early surgery patients, although postoperative length of stay did not differ significantly.

Discussion

The 48-hour threshold applied in this review is consistent with the admission-based definitions used in the included studies and broadly aligned with NICE and AAOS guideline principles [11,12]. Using this definition, 12.83% of patients were classified as receiving early surgery and 53.65% as delayed, while 33.53% (n = 43,250) could not be categorized because of incomplete reporting of surgical timing. This limits the precision with which timing-based comparisons can be interpreted.

Meta-Analysis Results

A sub-meta-analysis was conducted, including three retrospective observational studies comparing inpatient mortality between early and delayed fixation. The pooled sample comprised 53,575 patients, with 13,385 undergoing early surgery and 40,190 undergoing delayed intervention.

At the individual study level, effect estimates were inconsistent and imprecise in the smaller studies. Lizaur-Utrilla et al. [17] reported a RR of 0.50 (95% CI: 0.06-4.23), and Paul and Issac [18] reported an RR of 0.30 (95% CI: 0.07-1.30), both suggesting a potential reduction in mortality with early fixation, although with wide confidence intervals crossing unity. In contrast, Librero et al. [6], which contributed the majority of statistical weight (88.5%), reported a significantly increased risk associated with early surgery (RR: 2.10; 95% CI: 1.94-2.28).

The pooled analysis demonstrated a statistically significant association, with a combined RR of 1.99 (95% CI: 1.30-3.05; p = 0.001), indicating higher observed mortality in the early surgery group. Statistical heterogeneity was low to moderate (I² = 31%; χ² = 2.91), suggesting some variability between studies, although the overall estimate was largely driven by the dominant contribution of a single dataset.

These findings must be interpreted with caution. In Librero et al. [6], patients undergoing delayed surgery were older and had higher comorbidity burdens, as reflected by the Charlson comorbidity index and risk of malignancy index (RMI) scores, indicating a greater baseline risk of mortality. This raises the possibility of confounding by indication, whereby patients with poorer preoperative status are more likely to experience surgical delay. While delayed surgery is often associated with increased comorbidity burden and clinical instability, the opposite pattern observed in the dominant study suggests a potential imbalance in patient selection or unmeasured confounders. This raises the possibility of confounding by indication, whereby the timing of surgery reflects underlying patient status rather than acting as an independent determinant of outcome.

Furthermore, time-dependent biases, including immortal time bias, may have influenced the observed associations, as patients allocated to delayed surgery must survive long enough to undergo intervention. Variability in perioperative care pathways and institutional practices further complicates interpretation. Accordingly, although a statistically significant association was observed, the current evidence does not support a definitive causal relationship between time to surgery and inpatient mortality.

Narrative Interpretation of Results

Table 5 compares mortality outcomes reported by Lizaur-Utrilla et al. and Oakley et al. [17,19]. Oakley et al. found a significant association between earlier surgical intervention within 36 hours and reduced mortality rates, both at 30 days (6% vs. 21%) and at one year (28.6% vs. 42%), with p-values < 0.05 indicating statistical significance. In contrast, Lizaur-Utrilla et al., using a 48-hour threshold, found no significant differences in either three-month mortality (5% vs. 7.3%) or one-year mortality (14.4% vs. 13.6%), with p-values of 0.3777 and 0.799, respectively. These contrasting results suggest that early surgery may improve survival outcomes in some settings. However, the threshold used seems to be indeterminate and not a uniform practice. Conversely, Paul and Issac [18] reported a statistically significant threefold increase in in-hospital mortality associated with surgical delay (p = 0.01). Oakley et al. stated that, considering the lack of statistical significance of some of their findings, a better mortality rate may not necessarily result from a direct benefit of earlier surgery. The survival advantage recorded appeared to hinge less on racing the patient to the theater and more on meticulous preoperative optimization of cardiac, pulmonary, and metabolic comorbidities [19].

Lizaur-Utrilla et al. [17] revealed that delaying surgery up to four days does not inflate complication rates or imperil either short- or long-term mortality rates. Therefore, they argue that sufficient medical treatment optimization prior to surgery may be more important than adhering to a fixed universal timing of surgery. In contrast, Shoda et al. [21] resurrect the specter of delay, reporting that once the four-day threshold is crossed, inpatient mortality increases by more than 40%. Finally, although exact mortality rates were not reported in the study by Anthony et al. [22], the authors observed no discernible uptick in mortality despite surgical postponement, hinting that other, perhaps unmeasured, factors such as frailty indices, perioperative protocols, or postoperative mobilization may mask the effect of time itself.

Further results extracted from the pooled data, apart from mortality, included complications and length of stay. Daginnus et al. [20] reported that 94 patients experienced complications when surgery was performed within 24 hours, compared with 14 patients when surgery was delayed beyond 24 hours. Patczai et al. [23] reported a higher rate of non-prosthetic reoperations in internal fixation surgeries performed after a delay of more than 12 hours. Regarding hospital stay, Oakley et al. [19] found no statistically significant difference in the length of stay between early and delayed surgical groups (p = 0.236). Lizaur-Utrilla et al. [17] observed a shorter overall hospital stay in patients who underwent early surgery; however, the difference in postoperative length of stay between the groups was not statistically significant.

Limitations

This study is subject to several limitations that may have influenced the outcomes and interpretations. First, although there is significant clinical focus on the timing of surgical intervention for hip fractures, the existing body of literature on this specific topic remains relatively limited. Many studies addressing time to surgery often include subtrochanteric fractures within their datasets, thereby inadvertently introducing a distinct pathology that may demand different orthopedic management.

Second, surgical delays were not uniformly attributable to external or logistical constraints, such as limited operating room availability or staffing shortages. In multiple instances, postponement of surgery represented a deliberate clinical decision aimed at allowing sufficient time for the appropriate medical optimization of patients with complex comorbid conditions. However, these medically justified delays were not consistently differentiated from avoidable or system-related delays across the included studies, thereby introducing potential sources of bias and confounding into the interpretation and comparison of clinical outcomes.

Moreover, there was considerable inconsistency in how surgical timing cutoffs were defined across the included studies. While some authors adhered to the 48-hour window recommended by NICE guidelines [11], others employed different thresholds, including comparisons between surgeries performed within 24 hours, 72 hours, or even up to 96 hours post-admission.

This lack of uniformity in defining timeframes and reporting surgical timing not only complicated data extraction but also resulted in the exclusion of potentially valuable data. As a consequence, the overall completeness, comparability, and robustness of the pooled analysis were limited, thereby affecting the strength and clarity of the conclusions drawn from the review.

## Conclusions

This systematic review does not provide consistent evidence that earlier surgical intervention is associated with improved clinical outcomes in patients with intracapsular or intertrochanteric hip fractures. Although individual studies reported variable findings, the pooled analysis demonstrated a statistically significant association in the opposite direction, with higher observed inpatient mortality in the early surgery group. However, this result was largely driven by a single high-weight observational study, while smaller studies yielded imprecise and directionally inconsistent estimates.

Overall, the current evidence does not support a definitive causal relationship between surgical timing and clinical outcomes. Rather than indicating a clear benefit or harm, these findings reflect the ongoing uncertainty within the literature, highlighting the complexity of establishing a universal surgical timing threshold. In this context, a pragmatic, patient-centered approach that prioritizes timely intervention while allowing appropriate medical optimization remains essential in current clinical practice.
